# Aflatoxin contamination of human breast milk and complementary foods in southern Ethiopia

**DOI:** 10.1111/mcn.13081

**Published:** 2020-09-20

**Authors:** Mesfin Eshete, Samson Gebremedhin, Fikadu R. Alemayehu, Mestawet Taye, Bergene Boshe, Barbara J. Stoecker

**Affiliations:** ^1^ School of Nutrition, Food Science and Technology Hawassa University Hawassa Ethiopia; ^2^ School of Public Health Addis Ababa University Addis Ababa Ethiopia; ^3^ School of Animal and Range Sciences Hawassa University Hawassa Ethiopia; ^4^ College of Human Sciences Oklahoma State University Stillwater Oklahoma USA

**Keywords:** aflatoxin, breast milk, complementary foods, Ethiopia, mycotoxin

## Abstract

Exposure to unsafe level of aflatoxin in early life may lead to growth faltering. However, the extent of contamination of breast milk and complementary foods is poorly examined. We determined aflatoxin M_1_ (AFM_1_) and B_1_ (AFB_1_) contamination of human breast milk and cereal‐based cooked complementary foods, respectively, among households having children 6–23 months of age in Sidama zone, southern Ethiopia. Data were collected through two cross‐sectional surveys implemented in the wet (*n* = 180) and dry (*n* = 180) seasons. Eligible households (*n* = 360) were recruited from three agroecological zones (lowland, midland and highland, each with sample size of 120) using a multistage sampling technique. AFB_1_ and AFM_1_ levels were determined using enzyme‐linked immunosorbent assay. Mann–Whitney *U* and Kruskal–Wallis tests were performed to compare aflatoxin levels between seasons and across the agroecological zones. Among 360 breast milk samples tested, 64.4% had detectable AFM_1_ and 5.3% exceeded the 0.025 parts per billion (ppb) limit set by the European Union for infant milk. The median AFM_1_ in the lowlands was significantly higher than in the other agroecological settings (*P* < 0.001). By season, AFM_1_ was higher in breast milk samples collected in the dry season (*P* = 0.041). AFB_1_ was detected in 96.4% of the food samples tested, and 95.0% had concentration exceeding the permissible European Union limit of 0.1 ppb. The median AFB_1_ was significantly higher in the lowland (*P =* 0.002), but there was no difference between the seasons (*P* = 0.386). The study indicated that, in southern Ethiopia, foods intended for infants are heavily contaminated with AFB_1_. Contamination of breast milk is also a significant health concern.

**Key messages**

- In southern Ethiopia, complementary foods intended for infants and young children are heavily contaminated with AFB_1_.
- Contamination of breast milk with AFM_1_ is also a significant health concern.
- Lowland areas are more prone to aflatoxin contamination of breast milk and complementary foods as compared with the other agroecological zones.
- Breast milk contamination with AFM_1_ is higher during the dry season.

## 1 INTRODUCTION

Aflatoxins are a highly toxic group of mycotoxins produced by some species of the genus Aspergillus, including *Aspergillus flavus*, *Aspergillus parasiticus* and *Aspergillus nomius* (WHO and FAO, 2018). Several types of aflatoxins have been identified, yet four types (B_1_, B_2_, G_1_ and G_2_) and a secondary metabolite (M_1_) are regarded as significant contaminants of the food system (Williams et al., 2004). Foods frequently affected by aflatoxins include grains, nuts, oilseeds and spices stored under hot and humid conditions (WHO, 2018).

Aflatoxin exposure has serious adverse effects on human health (Williams et al., 2004). The relationship between chronic aflatoxicosis and liver cancer has long been established (IARC., 2002). Epidemiological and animal model studies have also proposed that high aflatoxin exposure may lead to growth faltering, immunity impairment, increased risk of infection and interference with micronutrient metabolism (Gong et al., 2004; Williams et al., 2004). In low‐ and middle‐income countries (LMIC), human exposure to unsafe levels of aflatoxin begins early in life through transplacental transfer as well as contaminated breast milk and complementary foods (Wild, 2007). In utero exposure may cause fetal and postnatal growth retardation, low birthweight and DNA methylation (Hernandez‐Vargas et al., 2015; Shuaib et al., 2010; Smith, Prendergast, Turner, Humphrey, & Stoltzfus, 2017; Turner et al., 2007), whereas exposure via foods and breast milk may lead to growth faltering including stunting (Magoha et al., 2014; Sadeghi et al., 2009). However, a recent systematic review of experimental and observational studies concluded that the existing evidence on the relationship between aflatoxin exposure and growth and immunity impairments in children is not adequate for any conclusive supposition (Tesfamariam et al., 2020).

Breast milk is widely regarded as the best source of nourishment for infants and offers numerous nutritional immunity and cognitive benefits (Horta & Victoria, 2013). Yet several harmful chemicals can contaminate breast milk and affect infants' health (Landrigan, Sonawane, Mattison, McCally, & Garg, 2002). In humans and animals consuming foods contaminated with aflatoxin B_1_ (AFB_1_)—the most potent carcinogen among the aflatoxin family—the toxin is converted into a secondary metabolite aflatoxin M_1_ (AFM_1_) and transferred into breast milk (Marchese et al., 2018). Though AFM_1_ is slightly less toxic than its precursor, the presence of AFM_1_ in breast milk is a significant concern for human health (Marchese et al., 2018). Studies conducted in LMIC suggested that considerable proportions of nursing mothers secrete high levels of AFM_1_ into their breast milk (Abdulrazzaq, Osman, Yousif, & Al‐Falahi, 2003; Fakhri et al., 2019; Jonsyn, Maxwell, & Hendrickse, 1995; Magoha et al., 2014; Oluwafemi, 2012; Polychronaki et al., 2006).

In LMIC, microbial contamination of complementary foods is common and is a major cause morbidity in young children (WHO, 2009). A growing body of literature also suggests that cereal‐based traditional and commercial foods designed for infants and young children are frequently contaminated with aflatoxin (Alamu et al., 2018; Ayelign, Woldegiorgis, Adish, & De Saeger, 2018; Blankson & Mill‐Robertson, 2016). A study conducted in four regions of Ethiopia that analysed complementary foods produced at the community level reported that aflatoxins were detected in most of the premilling, postproduction and stored samples (Ayelign et al., 2018).

The aim of this study is to evaluate AFB_1_ contamination of cereal‐based complementary foods intended for infants and young children and the AFM_1_ level in human breast milk and to assess the agroecological and seasonal variabilities of the exposure in Sidama zone, southern Ethiopia.

## 2 METHODS AND MATERIALS

### 2.1 Study design and setting

The study comprised two independent cross‐sectional surveys that were carried out in August 2017 (wet season) and March 2018 (dry season) in Sidama zone, southern Ethiopia. The survey sites were Hawassa Zuria, Dale and Hula districts and were purposely selected among the 19 districts of Sidama to represent the lowland (<1750 m above sea level (a.s.l)), midland (1750–2300 m a.s.l) and highland (>2300 m a.s.l) agroecological settings (Gebreselassie, Gase, & Deressa, 2013). All sites were visited during the two seasonal surveys.

Based on the projection of the 2007 National Census, in 2017, Sidama had a population size approaching four million (Population Census Commission [Ethiopia], 2008). The lowland areas of Sidama annually receive 400 to 800 mm of rain and exhibit mean annual temperature of 20°C–25°C. In the highlands, the annual rainfall is higher (1200–1600 mm) and the average annual temperature ranges between 15°C and 20°C. All the districts included in the surveys were predominately rural. The major staple diets in Sidama are maize and *Enset ventricosum.* *Enset ventricosum* commonly known as *Enset* or false banana is a drought‐resistant root crop, which is an important staple in south and southwestern Ethiopia.

### 2.2 Study subjects and eligibility criteria

We considered all mothers who were nursing and who had already introduced complementary foods to their children (6–23 months of age) eligible for the study. Nonbreastfeeding mothers and mothers who were yet to offer complementary foods to their infants were excluded with the intention of collecting both breast milk and complementary food samples from the same set of households. In the second survey, participants of the first survey were considered ineligible.

### 2.3 Sample size determination

In the two seasonal surveys, we included 360 eligible subjects. The sample size for each round (*n* = 180) was determined using G*Power 3.1 programme (Faul, Erdfelder, Lang, & Buchner, 2007) based on the assumption that the level of aflatoxin contamination of breast milk and complementary foods would be compared across the three agroecological zones using one‐way analysis of variance and between the two seasons using independent *t*‐test. The sample size estimation was made to detect 0.3 standardized mean difference (equivalent to medium effect size; Faul et al., 2007) between the two seasons and among three agroecological zones as a significant difference at 95% confidence level and 80% power.

### 2.4 Sampling approach

In both of the survey rounds, study participants were selected using a multistage cluster sampling approach. Initially, the total sample size (*n* = 180) for that survey was equally distributed to the three agroecological zones (60 per district). Then from each of the three districts, two *kebeles* (the smallest administrative unit in Ethiopia roughly having five thousand population) with the intended agroecological feature were randomly chosen. From each *kebele*, 30 subjects that fulfilled the inclusion criteria were selected using a simple random sampling approach. Prior to the data collection, study subjects that fulfilled the inclusion criteria were registered through a rapid listing, and the list was used as a sampling frame for the study. Few individuals who were not willing to take part in the study were replaced with eligible subjects from adjacent households.

### 2.5 Data collection tools and procedures

Trained enumerators administered the questionnaire and collected the samples. Information about the basic socio‐economic characteristics of the study participants, the feeding practices of the children and ingredients used for preparing the complementary foods were collected from the mothers of the index children using a structured and pretested questionnaire prepared in the local *Sidamu Afo* language. About 10 ml of breast milk was collected from each subject by manual self‐expression following standard procedures (UNEP, 2010). Additionally, about 100 g of cereal‐based cooked complementary food prepared for the index children was collected using a clean spoon and plastic bag. All the samples were transported in an icebox and kept frozen at −20°C until analysed.

### 2.6 Analysis of AFM_1_ and AFB_1_

We determined AFM_1_ and AFB_1_ levels using enzyme‐linked immunosorbent assay (ELISA) kits and reagents supplied by Helika (Helica Biosystems Inc., California). The limits of detection for the two tests were 5 pg/ml (parts per trillion [ppt]) and 0.2 ng/ml (parts per billion [ppb]), respectively. All the tests were made in duplicates, but spiked sample analysis was not done. Milk fat was first removed by centrifuging the milk samples in 10°C for 5 min at 2000 g. After removing the upper cream layer, the lower phase was used for quantitative testing. Assay protocol (#961AFLM01M‐96) provided by the manufacturer was followed (Helica Biosystems, Inc., 2020a).

For the AFB_1_ analysis, cereal‐based cooked complementary food samples including *Enset* products were first grounded to fine particle size. When different food samples were available from the same household, a composite sample with equal ratio was formed by uniform mixing of the ground samples. Concentration of AFB_1_ was measured according to the procedures (#941BAFL01B1‐96) provided by the manufacturer (Helica Biosystems, Inc., 2020b).

### 2.7 Data management and analysis

SPSS 20 software was used for data management and analyses. Prior to analysis, the AFM_1_ and AFB_1_ were screened for normality and outlier values. Both variables were skewed to the right and accordingly were analysed using nonparametric statistics. Prior to considering nonparametric tests, log transformation with base 10 and base e and square root transformation were attempted but did not yield normally distributed data. The median level of AFM_1_ and AFB_1_ concentrations in breast milk and food samples was compared across the three agroecological zones and the two seasons using Kruskal–Wallis and Mann–Whitney *U* tests, respectively. Post hoc analysis was performed to detect significance difference within the three agroecological zones. Proportion of breast milk and complementary food samples that exceeded the European Union (EU) thresholds of 0.025 and 0.1 ppb, set for infant milk and processed cereal‐based food including foods for infants and young children, respectively, were also determined (EU, 2006). The association between AFM_1_ and AFB_1_ concentrations in breast milk and food samples was analysed using Pearson correlation analysis.

### 2.8 Ethical considerations

Ethical clearance was secured from the Institutional Review Board of College of Medicine and Health Sciences, Hawassa University. The data were collected after taking written consent form the respondents. At the end of the study, all the respondents were given advice on how to prevent aflatoxin contamination of food.

## 3 RESULTS

### 3.1 Characteristics of the study participants

Across the two survey rounds and the three agroecological settings, a total of 360 lactating women having infants and young children 6–23 months of age were enrolled. The mean age (±SD) of the mothers was 26.3 (±4.4) years, and nearly two‐thirds (63.4%) were between 25 and 34 years of age. About a quarter (27.8%) had no formal education, and almost all were married. The median (IQR) household size was 5 (3–6), and one‐third had six or more family members. The median monthly household income was 20 (13–27) USD. The mean age of the infants and young children was 12.7 (±5.3) months, and 48.4% were between 6 and 11 months. Boys were slightly overrepresented at 52.2% (Table 1).

**TABLE 1:** Characteristics of the study participants, Sidama zone, southern Ethiopia, 2017–2018

Characteristics (*n* = 360)	Frequency	Percent
Age of mothers (in years)		
18–24	112	31.1
25–34	228	63.4
≥35	20	5.6
Marital status of the women		
Married	358	99.4
Divorced/separated	2	0.6
Educational status of respondents		
No formal education	100	27.8
Primary education 1st cycle (grades 1–4)	52	14.4
Primary education 2nd cycle (grades 5–8)	162	45.0
High school or above	46	12.8
Main source of household income		
Agriculture	294	81.7
Petty trade	24	6.7
Daily labour	17	4.7
Others	25	6.9
Household size		
2–3	98	27.2
4–5	134	37.2
6 or above	128	35.6
Age of child (in completed months)		
6–11	174	48.4
12–17	104	28.9
18–23	82	22.7
Sex of child		
Male	188	52.2
Female	172	47.8

### 3.2 Child feeding practice

All the children received breast milk on the day preceding the survey. The mean frequency of breastfeeding in the previous day was 9.1 (±3.7), and 64.2% were fed eight to 12 times. Most mothers (77.2%) introduced complementary foods to the index infants at 6 months of age. The most commonly used complementary foods were cereal‐based porridge (50.6%), flatbread (19.7%) and *Kocho* prepared from *Enset ventricosum* (12.2%). Among cereals maize, and among legumes broad bean and haricot bean, were predominately used for preparing complementary foods (Table 2).

**TABLE 2:** Feeding practice of the infant and young children in three agroecological settings of Sidama zone, southern Ethiopia, 2017–2018

Child feeding practice	Frequency	Percent
Frequency of breastfeeding in the previous day		
2–7	93	25.8
8–12	231	64.2
13 or above	36	10.0
Child's age at introduction of complementary food		
Before 6 months	26	7.2
At 6 months	278	77.2
After 6 months	56	15.6
Primary source for complementary foods		
Local market	147	41.2
Own agricultural production	56	15.7
Both	152	42.6
Donation	2	.6
The most commonly used complementary foods		
Porridge	182	50.6
Flatbread	71	19.7
*Kocho* (*Enset ventricosum*)	44	12.2
Bread	37	10.3
*Injera* (flatbread primary made out of *teff* flour)	23	6.4
Boiled cereals/legumes	3	0.8
Most commonly used cereals for preparing complementary foods		
Maize	320	88.9
Wheat	63	17.5
Barley	49	13.6
*Teff*	29	8.1
Sorghum	6	1.7
Most commonly used legumes for preparing complementary foods		
Broad bean	179	49.7
Haricot bean	150	41.7
Peas	78	21.7
Kidney bean	18	5.0

### 3.3 Aflatoxin M_1_ level of breast milk

The median (IQR) AFM_1_ concentration was 1.1 (undetectable to 4.4) ppt and ranged from undetectable to 143.3 ppt. Among 360 breast milk samples tested, 232 (64.4%) had detectable AFM_1_ and 19 (5.3%) exceeded the EU threshold of 25 ppt set for infant milk and related products (EU, 2006). Table 3 compares the median AFM_1_ across the three agroecological zones and the two seasons. AFM_1_ was significantly higher in the lowlands than in the other agroecological settings (*P* < 0.001). Seasonally, AFM_1_ was significantly higher in samples collected during the dry season (*P* = 0.041) (Table 3).

**TABLE 3:** AFM_1_ concentration of breast milk and proportion of samples with detectable AFM_1_ in three agroecological settings and two seasons of Sidama zone, August 2017 and March 2018

Factor (*n* = 360)	Detectable		Median (IQR) (ppt)	*P* value
	Frequency	%		
Agroecology				
Lowland (*n* = 120)	101	84.2	2.55 (0.86–9.28)	<0.001^*^
Midland (*n* = 120)	58	48.3	0.97 (0.00–1.89)	
Highland (*n* = 120)	73	60.8	0.61 (0.00–7.85)	
Season				
Wet (*n* = 180)	115	63.9	0.94 (0.00–2.86)	0.041^*^
Dry (*n* = 180)	117	65.0	1.18 (0.00–9.96)	

^*^ Statistically significant difference among the median values at 5% level of significance.

Among breast milk samples collected from the highland area, 10 (8.3%) exceeded the maximum AFM_1_ tolerable limit. Similarly, five (4.2%) samples from the midland and four (3.3%) from the lowland exceeded this limit. By seasons, five (2.8%) of the samples collected in the wet season and 14 (7.8%) collected in the dry season exceeded the threshold.

### 3.4 Aflatoxin B_1_ level of cereal‐based complementary food

The median (IQR) AFB_1_ level in the 360 cereal‐based complementary food samples tested was 0.8 (0.5–1.2) ppb. AFB_1_ was detected in nearly all, 347 (96.4%), of the samples, and in 342 (95.0%) of the cases, the concentration exceeded the permissible limit of 0.1 ppb set for processed cereal‐based foods designed for infants and young children (EU, 2006). The median AFB_1_ was significantly higher in the lowland (*P* = 0.002), but there was no significant difference between the two seasons (*P* = 0.386) (Table 4).

**TABLE 4:** Concentration of AFB_1_ in complementary foods and proportion of samples with detectable AFB_1_ in three agroecological settings and two seasons of Sidama zone, August 2017 and March 2018

Factor (*n* = 360)	Detectable		Median (IQR) (ppb)	*P* value
	Frequency	%		
Agroecology				
Lowland (*n* = 120)	117	97.5	0.95 (0.61–1.47)	0.002^*^
Midland (*n* = 120)	111	92.5	0.77 (0.44–1.07)	
Highland (*n* = 120)	119	99.2	0.72 (0.48–1.17)	
Season				
Wet (*n* = 180)	175	97.2	0.79 (0.55–1.10)	0.386
Dry (*n* = 180)	172	95.6	0.80 (0.49–1.34)	

^*^ Statistically significant difference among the median values at 5% level of significance.

Among food samples collected from the lowland, midland and highland districts, 96.7%, 91.7% and 96.7%, respectively, had AFB_1_ concentration that exceeded the tolerable EU limit (0.1 ppb). Season‐wise, 96.1% of samples collected in wet and 93.9% in the dry season exceeded this threshold.

Correlation analysis between AFM_1_ in breast milk and AFB_1_ in complementary food samples showed statistically significant but weak relationship (*r* = 0.146, *P* = 0.006).

## 4 DISCUSSION

The study provided an overview of AFM_1_ and AFB_1_ contamination of human breast milk and complementary foods in southern Ethiopia. We found that cereal‐based foods intended for infants and young children are heavily contaminated with AFB_1_. Furthermore, considerable contamination of breast milk, representing exposure of the mothers and their nursing infants to unsafe level of aflatoxin, was identified. In general, contamination of breast milk and complementary foods was more common in the lowland area, and breast milk was more frequently contaminated during the dry season.

Among breast milk samples tested, 64% had detectable AFM_1_, whereas 5% exceeded the EU threshold set for infant milk. Previous studies from the sub‐Saharan Africa (SSA) have reported inconsistent findings. A study from Nigeria found that AFM_1_ was detected in 14% of the samples (Oluwafemi, 2012). In northern Tanzania, Magoha et al. (2014) identified extremely high contamination whereby 90% of the samples exceeded the aforementioned AFM_1_ EU threshold. In another study from Nigeria, 78% had detectable AFM_1_ and 38% exceeded the tolerable limit (Anthony et al., 2016). A meta‐analysis concluded that AFM_1_ levels in human breast milk demonstrated pronounced variation across different regions of the world, and in Africa, nearly half of the samples had detectable AFM_1_ (Fakhri et al., 2019). In general, parallel to the findings of the other studies conducted in the SSA region, our study suggested that aflatoxin contamination of breast milk is of significant health concern in southern Ethiopia.

Aflatoxin contamination of complementary foods appears to be high in Sidama, as AFB_1_ was detected in nearly all samples and the concentration exceeded the EU threshold in 95% of the cases. In 20 districts of Ethiopia involved in UNICEF‐supported community‐based production of complementary foods, Ayelign et al. (2018) observed that aflatoxins were detected in nearly all of premilling, postproduction and stored complementary foods. Yet only 2% of the samples exceeded the limit of 0.1 ppb. As compared with our study, the lower proportion of samples that exceeded the limit can be due the better hygienic practices implemented at community‐based complementary food production centres. At the centres, food was stored in improved grain banks and food handles were trained on approaches for preventing contamination of complementary food ingredients (Ayelign et al., 2018).

High aflatoxin contamination of complementary foods has also been reported in other SSA countries. A study from the greater Accra region of Ghana found that 71% of the processed foods contained AFB_1_ higher than the EU permissible limits (Blankson & Mill‐Robertson, 2016). Among commercially produced complementary foods from Dar es Salaam and Arusha cities, Tanzania, aflatoxin was detected in 60% of the samples and 30% exceeded the tolerable limit (Rushunju, Laswai, Ngowi, & Katalambula, 2013). High aflatoxin in home or commercially produced complementary foods in Ethiopia and other African countries can be explained by multiple reasons including favourable hot and humid conditions and unhygienic food storage and processing practice (Achaglinkame, Opoku, & Amagloh, 2017). A study from Ethiopia also suggested that mothers had suboptimal knowledge about aflatoxin and frequently practiced poor food storage and processing methods that favour mould growth (Beyene, Woldegiorgis, Adish, De Saeger, & Tolossa, 2016).

We observed that AFM_1_ and AFB_1_ contaminations of breast milk and complementary foods were more frequent in the lowland than in the mid and highlands. This is consistent with the understanding that warm climatic situations favour mould growth and aflatoxin contamination of the food system (Achaglinkame et al., 2017). Kachapulula, Akello, Bandyopadhyay, and Cotty (2017) also concluded that aflatoxin contaminations of maize and groundnut samples were higher in the lowlands than in the other agroecological zones of Zambia. The finding may suggest that aflatoxin contamination prevention activities need to target lowland areas.

In this study, the AFM_1_ contamination of breast milk appears to be higher during the dry season. This is compatible with the knowledge that hot climate facilitates the proliferation of mould in the food system. Previous studies have also reported comparable seasonal variations. In Lebanon, according to Elaridi, Bassil, Kharma, Daou, and Hassan (2017), AFM_1_ was significantly higher in spring and summer than in fall and winter seasons. Kılıç Altun, Gürbüz, and Ayağ (2017) also reported that, in Turkey, a statistically significant difference in AFM_1_ was observed between samples collected in December and June.

We observed a significant correlation between AFM_1_ in breast milk and AFB_1_ in complementary food. The relationship is likely the reflection of the level of contamination of household food supply with aflatoxin.

While interpreting the findings of our study, the strengths and limitations should be taken into consideration. A strength is that we represented multiple agroecological zones and seasons and compared the level of aflatoxin contamination. We also enrolled a large sample size and randomly selected study participants through community‐based surveys to make the findings more generalizable to the area. Further, we tested for both AFM_1_ and AFB_1_ contamination, which are highly relevant to infants and young children.

Conversely, the study has some limitations. The comparison across seasons and ecological settings could have been confounded by extraneous factors including type and duration of food storage that were not measured in the study. The fact that comparison of aflatoxin across seasons and agroecology was made using nonparametric tests could compromise the power of the analyses. Furthermore, despite using multistage sampling procedure, we did not correct the sample size using design effect, and this might have further compromised the power of the study. Finally, as there is no globally agreed aflatoxin limit for breast milk, we rather used the limit set for infant milk and formulae.

## 5 CONCLUSION

The study demonstrated that in Sidama zone, southern Ethiopia, foods intended for infants and young children are heavily contaminated with AFB_1_. AFM_1_ contamination of breast milk is also a significant health concern suggesting exposure of mothers and their infants to aflatoxin. In general, aflatoxin contamination of breast milk and complementary foods is more common in the lowland area, and breast milk contamination is more frequent during the dry season. We recommend that comprehensive preharvest and postharvest aflatoxin contamination prevention activities, including promotion of good agricultural practices, safe crop harvesting, storage and processing techniques, be implemented in Sidama, especially in the lowland areas.

## CONFLICTS OF INTEREST

The authors declare that they have no conflict of interest in the findings of the study.

## CONTRIBUTIONS

SG, BJS, MT and FR designed the study. ME and BB performed the research and conducted the lab analysis. ME, FR and SG analysed the data. ME and SG wrote the paper.
